# Matrix-Immobilized BMP-2 on Microcontact Printed Fibronectin as an *in vitro* Tool to Study BMP-Mediated Signaling and Cell Migration

**DOI:** 10.3389/fbioe.2015.00062

**Published:** 2015-05-11

**Authors:** Kristin Hauff, Chiara Zambarda, Miriam Dietrich, Maria Halbig, Anna Luise Grab, Rebecca Medda, Elisabetta Ada Cavalcanti-Adam

**Affiliations:** ^1^Department of Biophysical Chemistry, Institute of Physical Chemistry, University of Heidelberg, Heidelberg, Germany; ^2^Applied Chemistry, University of Reutlingen, Reutlingen, Germany; ^3^Department of New Materials and Biosystems, Max Planck Institute for Intelligent Systems, Stuttgart, Germany

**Keywords:** BMP-2, fibronectin, microcontact printing, C2C12 myoblasts, BMP/Smad signaling

## Abstract

During development, growth factors (GFs) such as bone morphogenetic proteins (BMPs) exert important functions in several tissues by regulating signaling for cell differentiation and migration. *In vivo*, the extracellular matrix (ECM) not only provides support for adherent cells, but also acts as reservoir of GFs. Several constituents of the ECM provide adhesive cues, which serve as binding sites for cell trans-membrane receptors, such as integrins. In conveying adhesion-mediated signaling to the intracellular compartment, integrins do not function alone but rather crosstalk and cooperate with other receptors, such as GF receptors. Here, we present a strategy for the immobilization of BMP-2 onto cellular fibronectin (cFN), a key protein of the ECM, to investigate GF-mediated signaling and migration. Following biotinylation, BMP-2 was linked to biotinylated cFN using NeutrAvidin as cross-linker. Characterization with quartz crystal microbalance with dissipation monitoring and enzyme-linked immunosorbent assay confirmed the efficient immobilization of BMP-2 on cFN over a period of 24 h. To validate the bioactivity of matrix-immobilized BMP-2 (iBMP-2), we investigated short- and long-term responses of C2C12 myoblasts, which are an established *in vitro* model for BMP-2 signaling, in comparison to soluble BMP-2 (sBMP-2) or in absence of GFs. Similarly to sBMP-2, iBMP-2 triggered Smad 1/5 phosphorylation and translocation of the complex to the nucleus, corresponding to the activation of BMP-mediated Smad-dependent pathway. Additionally, successful suppression of myotube formation was observed after 6 days in sBMP-2 and iBMP-2. We next implemented this approach in the fabrication of cFN micropatterned stripes by soft lithography. These stripes allowed cell-surface interaction only on the patterned cFN, since the surface in between was passivated, thus serving as platform for studies on directed cell migration. During a 10-h observation time, the migratory behavior, especially the cells’ net displacement, was increased in presence of BMP-2. As such, this versatile tool retains the bioactivity of GFs and allows the presentation of ECM adhesive cues.

## Introduction

The use of growth factors (GFs), such as bone/body morphogenetic proteins (BMPs), in biomedical applications is gaining importance over the last few years (Crouzier et al., 2011; Kang et al., 2011). The lack of control over the amount and release of GFs often leads to unwanted ectopic side effects (Cheung and Phillips, 2006; Carragee et al., 2011). Especially BMP-2 is already used for clinical applications, however, ectopic bone formation has been observed (Rosen, 2009; Luca et al., 2010). The immobilization of the GFs not only reduces ectopic side effects but also proves to be more cost-efficient, since lower amounts are required to obtain the desired local effect (Igwe et al., 2012), while allowing for sustained presentation. Current challenges in GF immobilization strategies are represented by (i) achieving control over the amount of the immobilized molecule and (ii) avoiding hindrance and interference with GF bioactivity (Almodóvar et al., 2014).

Bone morphogenetic proteins were originally investigated for their ability to regulate the formation of new bone. At the cellular level, BMPs direct and participate in different processes like cell growth, apoptosis, differentiation, and migration (Sotobori et al., 2006). BMPs bind the heteromeric receptor complexes composed of type I and type II trans-membrane serine/threonine kinase receptors (Miyazono et al., 2005; Sieber et al., 2009). The activation of BMP receptors follows two different routes: activation through preformed receptor complexes triggers a Smad-dependent pathway, while complexes formed upon BMP binding, the so-called BMP-induced signaling complexes, initiate the activation of Smad-independent, p38-dependent pathways (Nohe, 2001). The Smad-dependent signaling pathway is mediated by endocytosis through functional clathrin-coated pits, while Smad-independent pathways rely on the association of caveolae with the receptor complexes (Hartung et al., 2006). Nevertheless, recent studies on the covalent immobilization of BMP-2 on surfaces indicated that BMP-2 internalization is not necessary to trigger Smad 1/5 phosphorylation, suggesting that BMP-dependent pathways might be already activated by GF binding to the receptors (Pohl et al., 2012). Concerning the role of BMP in cell migration, Smad-independent pathways have been observed during BMP-2-mediated migratory effects. It has been reported that BMP-2 can activate Cdc42/PAK/LIMK and p38/MK2/Hsp25 pathways independently (Gamell et al., 2008).

The extracellular matrix (ECM) is a meshwork of glycoproteins and glycosaminoglycans, which provide structural and functional integrity to tissues and organs. Amongst them, proteins such as collagens and elastin, and glycoproteins such as fibronectin (FN) are secreted and arranged in a fibrillar network by various cell types, e.g., fibroblasts. FN is an abundant protein that exists in two isoforms derived by alternative splicing: the soluble plasma FN circulating in the blood and the insoluble cellular fibronectin (cFN), which is a component of the ECM. The interactions between cells and FN are mediated by the cell-binding motif RGD (arginine–glycine–aspartate) present in FN (Hynes, 2009).

The ECM not only offers a support for adherent cells and regulates cell migration and differentiation (Yamaguchi et al., 2005), but also presents a reservoir of GFs, which influence cell behavior (Folkman et al., 1988; Taipale and Keski-Oja, 1997; Hynes, 2009). Proteoglycans, for example, bind fibroblast growth factors (FGFs) and vascular endothelial growth factors (VEGFs) through their heparin chains (Hynes, 2009). ECM proteins that do not contain such a pronounced sugar moiety are also able to interact with GFs. For instance, FN, vitronectin, and collagen type II have been shown to bind insulin-like GF-binding protein 5, hepatocyte GF, and transforming growth factor-β (TGF-β) through specific binding sites, respectively (Xu et al., 2004; Hynes, 2009). How GFs transduce information from the matrix to the cells has not yet been conclusively clarified. Different processes may occur for different GFs. One possibility is that GFs interact with their receptors while they are bound to the matrix and the ECM proteins act like cofactors. The release of GFs upon degradation of the ECM due to injury or proteolytic activity is also possible (Hynes, 2009). This results in the availability of GFs that can interact with their receptors at the cell membrane independent of matrix components. This presentation mode offers a spatially and temporarily defined stimulation with soluble GFs. Among them, it is known that the cells secrete TGF-β as latency-associated peptide and it interacts with fibrillins, incorporating into the ECM. Proteolytic degradation or mechanical stretching by trans-membrane integrins releases active TGF-β molecules that bind to their respective TGF-β receptors (Horiguchi et al., 2012).

Several approaches have been developed to immobilize GFs to ECM components. For the delivery of BMP-2, new ECM biomimetic systems have been reported, like inkjet printing of BMP-2 patterns on fibrin substrates (Phillippi et al., 2008), soft biopolymeric films presenting BMP-2 and hyaluronan hydrogels (Patterson et al., 2010; Crouzier et al., 2011).

Combining the presentation of GFs with adhesive matrix molecules offers the possibility to investigate the signaling and crosstalk of different pathways involved in cell adhesion and migration. An approach to better define, engineer, and analyze cell behavior is the fabrication of micropatterned adhesive substrates, which present spatially defined ECM proteins surrounded by non-adhesive molecules to backfill the area in between (Jackman et al., 1999; Folch and Toner, 2000; Whitesides et al., 2001; Zheng et al., 2012). Additionally, to assess and quantify complex cellular processes such as cell migration, micropatterned stripes serve as excellent substrates to determine migration speed and persistence (Petrie et al., 2009; Vedula et al., 2012; Kasten et al., 2014).

In this study, we use microcontact printing to manufacture FN-coated surfaces (Csucs et al., 2003), which are further functionalized with immobilized BMP-2 (iBMP-2) molecules. We report on the influence of matrix-iBMP-2 on cell behavior. We chose C2C12 myoblasts as *in vitro* system, since this cell line is an established model for BMP-2 signaling. These cells fuse and form contractile myotubes in the absence of BMP-2, but commit to the osteogenic lineage upon BMP-2 exposure (Katagiri et al., 1994). The phosphorylation and translocation of certain Smad proteins, namely Smad 1/5/8, can be used as short-time read-out signal to determine the bioactivity of BMP-2 (Sieber et al., 2009; Bragdon et al., 2011).

## Materials and Methods

### Preparation of protein solutions

Cellular fibronectin from human foreskin fibroblasts (Sigma-Aldrich, St. Louis, MO, USA) was dissolved in sterile water (1 mg/ml) and dialyzed against PBS overnight using Dispo Biodialyzer (MWCO 5 kDa, Sigma-Aldrich). Recombinant human BMP-2 (rhBMP-2) derived from *E. coli* or *CHO* cells (355-BEC/CF, 355-BM/CF, R&D Systems, Minneapolis, MN, USA) was reconstituted in 1 M NaCl (99.5% p.a. ACS ISO, Carl Roth, Karlsruhe) in PBS to a concentration of 0.1 mg/ml. NeutrAvidin (NA; A2666, Life Technologies, Eugene, OR, USA) was dissolved in PBS (5 mg/ml).

### Biotinylation of cFN and BMP-2

For protein biotinylation at free amines on the lysine side chains, EZ-Link^®^ NHS-PEG 12-biotin (Thermo Scientific, Rockford, IL, USA) was dissolved in dry dimethyl sulfoxide (DMSO, Merck, Darmstadt) to a concentration of 25 mM. For labeling cFN, a 100-fold molar excess of the linker was added to the protein. BMP-2 was labeled by adding a molar excess of 40, respectively. Afterwards, the solutions were incubated at room temperature (RT) for 1 h while shaking. Biotinylated cFN (cFN-biotin) or BMP-2 (BMP-2-biotin) was purified overnight by dialysis against PBS or 1 M NaCl in PBS, respectively.

### Fluorescent conjugation of cFN

To visualize the micro patterned structures, cFN was fluorescently labeled. Atto-647N-NHS dye (ATTO-TEC GmbH, Siegen, Germany) was dissolved in dry DMSO (10 mg/ml) and added to the cFN solution at a molar excess of 50. The solution was incubated for 1 h while shaking. To remove unconjugated fluorophores, the mixture was dialyzed overnight against PBS yielding fluorescently labeled cFN (cFN-647).

### Photolithography and microcontact printing

Photolithography was used to produce the stamp master. Besides the silanization step, all production steps were performed in a clean room. A silicon wafer (Silicon Materials, Kaufering, Germany) with a diameter of 5 cm was spin coated using the negative photoresist SU-8 2002 (Microchem Corp., Newton, MA, USA) according to the manufacturer’s instructions, yielding a thickness of 2.7 μm. Then, a soft bake at 65°C for 2 min and at 95°C for another 2 min was performed. Contact exposure through a chrome mask, aligned by a custom-made vacuum mask holder, was achieved with UV light (exposure time 1.9 s). Here, a mask with a line width of 20 μm and spacing of 50 μm was used. Following post-baking at 65°C for 3 min, the resist was developed in mr-DEV 600 developer (micro resist technology GmbH, Berlin) for 75 s. The stamp master was dried in a stream of nitrogen and silanized with 1H1H2H2H-perfluorooctyltrichlorosilane (ABCR GmbH & Co. KG, Karlsruhe, Germany) in a desiccator for 6 h.

Polydimethylsiloxane (PDMS) (Sylgard 184 Silicon Elastomer Kit, Dow Corning, Seneffe, Belgium) was prepared according to the manufacturer’s instructions and degassed under vacuum. One milliliter of PDMS was poured into a Petri dish containing the stamp master, degassed in vacuum, and subsequently cured at 65°C overnight. Afterwards, the PDMS was detached from the stamp master, cut into 1 cm^2^ stamps, incubated in *n*-hexane for 30 min, and sonicated in *n*-hexane for 5 min to remove unreacted monomers or crosslinkers.

**Figure 1:**
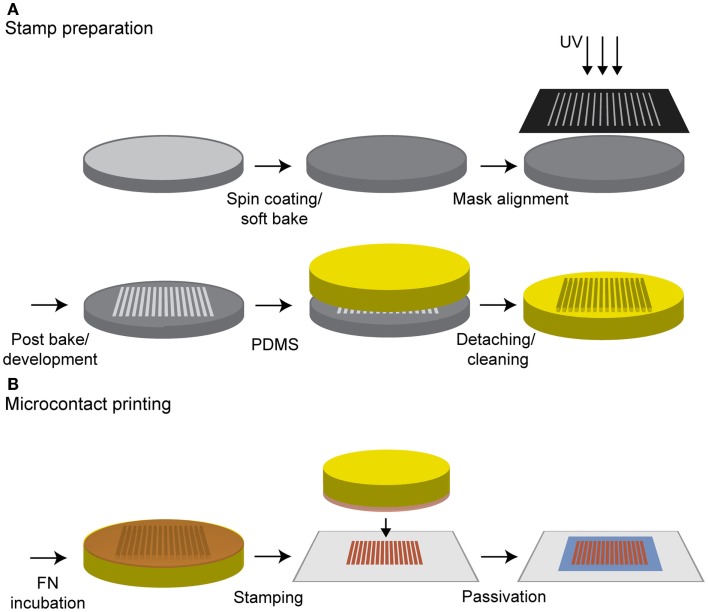
**Procedure for microcontact printing**. **(A)** A silicon wafer was spin coated with a negative photoresist and treated according the manufacturer’s instructions. Contact exposure through a chrome mask with UV light and further processing resulted in the stamp master. PDMS was poured on it, cured in an oven and was ready to use after a cleaning step in *n*-hexane. **(B)** The stamp was incubated with a cFN solution and the protein was adsorbed on the stamp surface. After a washing step, the stamp was dried, inverted, and gently pressed on an oxygen plasma-treated glass substrate whereby the protein structure was transferred to the glass. A final incubation with PLL–PEG passivated the surface in between the protein structure to prevent unspecific cell adhesion.

For microcontact printing, the stamp surface was coated with a mixture of 5 μl of cFN solution and 45 μl of PBS to obtain a final concentration of 0.1 mg/ml. As an alternative to the striped structure, a homogeneous PDMS stamp was applied to produce surfaces homogeneously covered with cFN. The solution was incubated for 40 min, after which the excess cFN was removed. To minimize the amount of labeled cFN, a mixture consisting of a ratio of 9:1 was incubated on the homogenous stamps with an area of 1cm^2^. Glass coverslips (Carl Roth, Karlsruhe, Germany) were cleaned with ethanol in an ultrasonic bath for 15 min. Before use, the glass slides were dried under a stream of nitrogen and treated with oxygen plasma [GigaEtch, PVA TePla, Kirchheim (München), Germany] at 0.4 mbar and 150 W for 10 min. The stamps were washed twice with 100 μl PBS, dried, and immediately placed onto the glass for 30 s. The stamps were carefully removed from the coverslip and rinsed with PBS several times. To prevent unspecific cell adhesion between the stripes, the cFN-patterned surfaces were incubated with 100 μl of 0.1 mg/ml poly-l-lysine-*g*-poly (ethylene glycol) [PLL (20 kDa)-g(3.5)–PEG (2 kDa), PLL–PEG, Surface Solutions, Switzerland] in HEPES buffer for 30 min (see Figure 1). To minimize the amount of PLL–PEG per substrate, a piece of parafilm was used to ensure an even coverage. Samples were then washed with PBS.

For soluble BMP-2 (sBMP-2), cFN and cFN-647 were added in equal amount, while for the iBMP-2, the biotinylated cFN (cFN-biotin) was used. For the immobilization of BMP-2, the cFN-biotin micropatterned surfaces were incubated with NA at a concentration of 0.01 mg/ml in PBS for 30 min followed by washing with PBS. Afterwards, 50 μl of BMP-2-biotin in PBS with a final concentration of 0.01 μg/μl was added for 1 h. Since the stamped surfaces have an area of approximately 1 cm^2^, the maximal possible surface concentration was 0.5 μg/cm^2^. The biotin linker consists of a 56 Å PEG spacer that mediates the immobilization of BMP-2 to NA and at the same time allows for a certain degree of mobility due to its length.

### Quartz crystal microbalance with dissipation monitoring

The interaction between cFN-biotin, NA, and BMP-2-biotin from *E. coli* cells was investigated using a quartz crystal microbalance with dissipation monitoring (QCM-D). All QCM-D experiments were performed in an E4 QCM-D from Q-Sense (Vaestra Froelunda, Sweden) using open modules. The sensing elements were silica-coated QCM-D crystals (QSX 303, Vaestra Froelunda, Sweden) with a fundamental frequency of 4.95 MHz. FN was microcontact printed on the activated sensor using a flat stamp as described in Section “Photolithography and Microcontact Printing.” The passivation step was not necessary as only sensors with a homogeneous layer of FN were prepared and the surfaces were not used for cell experiments. Unspecific protein adsorption was determined by direct comparison of adsorption processes on sensors with and without NA-binding sides, e.g., for each measurement, two sensors were functionalized with cFN and two sensors were coated with cFN-biotin. All samples were measured in real-time under the same conditions. They were incubated simultaneously and under the same conditions in NA and washed before mounting the crystals in the QCM-D machine. The temperature was adjusted to laboratory conditions and the stable oscillation of the sensor was validated. Two-hundred microliters of PBS were added in each chamber. The baseline was detected for a few minutes. Next, the buffer was exchanged by 200 μl of BMP-2-biotin (5 μg/cm^2^) for 1 h and afterwards the sensors were washed with PBS until a final plateau was reached.

### Enzyme-linked immunosorbent assay

An enzyme-linked immunosorbent assay (ELISA) kit (DuoSet ELISA, R&D Systems) was used to detect the BMP-2-biotin release. Homogeneously stamped cFN-biotin surfaces were prepared as described in Section “Photolithography and Microcontact Printing,” in the presence of cFN-biotin or cFN without biotinylation as negative control. The passivation step was omitted, as in the QCM-D characterization. After incubation with BMP-2-biotin *CHO*, the solutions containing the unbound proteins were collected as well as the two washing solutions and the overnight washing solutions at 4°C. The samples were treated according to the manufacturer’s instructions. In brief, the 96-well microplate was coated with capture antibody (part#840968 of DuoSet ELISA, R&D Systems) and the samples were incubated after a blocking step in reagent diluent (1% BSA in PBS, pH 7.2–7.4, 0.2 μm filtered). The detection antibody system [consisting of biotinylated anti-BMP-2 mouse IgG and streptavidin horseradish peroxidase (HRP)] was replaced with BMP-2 antibody directly coupled to HRP (part#892142 of Quantikine, BMP-2 Immunoassay, R&D Systems) as it would interfere with the detection of BMP-2-biotin. The absorbance was measured at 450 nm with a reference point at 570 using a plate reader (Infinite M200, Tecan, Männedorf, Switzerland). All values were corrected by the zero standard. Plotting the common logarithm of the absorbance of averaged standard samples against the common logarithm of the concentration generates a linear standard curve, which was used for quantification of BMP-2 concentrations in solution.

### Cell culture

Mouse C2C12 myoblasts (ATCC CRL-1772) were cultured as sub-confluent monolayers in Dulbecco’s modified Eagle’s medium containing 4.5 mg/ml glucose, 4 mM l-glutamine, and 1 mM sodium pyruvate (DMEM, 41966-029, Gibco Life Technologies, Carlsbad, CA, USA) supplemented with 10% (v/v) fetal bovine serum (FBS, S0115m, Biochrom AG, Berlin, Germany) and 1% (v/v) penicillin/streptomycin (15140, Gibco) at 37°C and 5% CO_2_.

### Indirect immunofluorescent staining and microscopy

The translocation of the Smad complex into the nucleus was analyzed to investigate the short-term effect of BMP-2 *E. coli* on myoblasts. Following serum starvation overnight, C2C12 cells were seeded on homogeneously stamped cFN surfaces at a density of 10,000 cells were seeded on a 20 mm × 20 mm glass coverslip and incubated for 30 min to allow adhesion on the substrates. Thereafter, 0.5 μg of BMP-2 was added to the medium (sBMP-2), which was considered time point 0. All samples were fixed after different time points (*t*_0_, 10, 30, 45, 60, 90 min) with 4% (w/v) paraformaldehyde (PFA) in PBS for 30 min, rinsed with PBS, and permeabilized with 0.1% (v/v) Triton-X-100 in PBS (Sigma-Aldrich) for 5 min. After blocking of unspecific binding sites with 1% (w/v) BSA in PBS (1% BSA) for 30 min, the surfaces were incubated with the primary antibody anti-p-Smad 1/5 rabbit IgG (S463/465, clone 41D10, 9516S, Cell Signaling, Danvers, MA, USA) at a dilution of 1:50 (according to the manufacturer’s recommendation) in 1% BSA at RT for 1 h. After washing twice with 1% BSA for 10 min, the samples were incubated with the respective secondary antibody Alexa Fluor 488^®^ goat anti-rabbit IgG (Life technologies, A11034) at a final concentration of 5 μg/ml in 1% BSA as well as with Phalloidin-TRITC (0.2 μg/ml) at RT for 1 h and washed again twice with 1% BSA for 10 min. Finally, the samples were embedded in Mowiol (Sigma-Aldrich) containing 0.1% (v/v) DAPI for an additional staining of the nucleus. Stained samples were imaged with an upright wide field microscope (Leica DM6000B, Leica Microsystems GmbH, Wetzlar, Germany) using a 40× (HCX PL APO 40×/0.85 CORR) objective lens. Image processing was done using ImageJ software (version 1.48c, Rasband, W.S., ImageJ, U.S. National Institutes of Health, Bethesda, MD, USA)^1^. For analysis, a binary image of the nucleus and the cytosol was produced. The obtained masks were used to quantify the pSmad signal in these areas. To calculate the integrated densities, the area was multiplied by the mean gray value. In addition, the integrated density of the cytosol was divided by the integrated density of the nucleus.

Since the presence of BMP-2 suppresses the differentiation of C2C12 cells into myotubes and promotes osteogenic differentiation, the myogenic phenotype was assessed by staining for myosin heavy chain (MHC) in confluent cell monolayers. C2C12 cells were serum-starved overnight and cultured at a density of 5,000 cells/cm^2^ on homogeneous stamped cFN-biotin surfaces under high serum conditions for 6 days at 37°C and 5% CO_2_. Afterwards, cells were fixed and stained with anti-MHC mouse IgG (MF20, DSHB, Iowa City, IA, USA) at a concentration of 2.3 μg/ml followed by Alexa Fluor^®^ 488 goat anti-mouse IgG (Life Technologies, A11001) and DAPI.

### Western blot

Short-term response was evaluated by western blot detection of phosphorylated Smad 1/5. C2C12 cells were serum-starved overnight and 10,000 cells for each sample were seeded for 30 min at 37°C and 5% CO_2_ at a 1 cm^2^ homogeneously stamped cFN surface with or without BMP-2 *E. coli*, as described in Section “Enzyme-Linked Immunosorbent Assay.” After different time points (0, 30, 45, 60 min), cells were lysed in 2× Laemmli Buffer (4% SDS, 20% Glycerol, 120 mM Tris–HCl, pH 6.8, 200 mM DTT, 0.02% Bromphenolblue) at −80°C overnight and boiled at 95°C for 5 min. The total proteins homogenates were loaded onto SDS-PAGE NuPAGE 4–12% Bis–Tris Gel, NuPAGE MES Running Buffer (Novex, Life Technologies, Carlsbad, CA, USA) and blotted into nitrocellulose membrane (GE Healthcare, Little Chalfont, UK) in NuPAGE Transfer Buffer (Novex, Life Technologies, Carlsbad, CA, USA). The blots were blocked with 3% milk (Carl Roth, Karlsruhe, Germany) in Tris-buffered saline with 0.1% Tween-20 (TBST, 50 nM Tris, pH 8.0, 150 mM NaCl, 0.1% Tween-20) at RT for 1 h and incubated at RT overnight in TBST 1% milk 1/1000 rabbit anti pSmad 1/5 (#9516, Cell Signaling, Danvers, MA, USA) or 1 h in TBST 1% milk 1/2000 mouse anti β-actin (A1978, Sigma-Aldrich, St. Louis, MO, USA). Blots were then washed in TBST and incubated for 1 h in TBST 1% milk at RT with HRP-conjugated secondary antibodies 1:5000 goat anti-rabbit or goat anti-mouse (Santa Cruz Biotechnology, Heidelberg, Germany). After washing, the membranes were incubated in ECL prime western blotting detection reagent (GE Healthcare, Little Chalfont, UK) and chemiluminescence detected using a LAS 3000 (Fujifilm, Tokyo, Japan).

### Time-lapse video microscopy and analysis of cell migration

Due to the dimension of the stripes, the cells were restricted to only migrate in a linear manner. For migration experiments, C2C12 cells were serum-starved overnight. Ten thousand cells were seeded in 2 ml medium at 37°C and 5% CO_2_ on 20 mm × 20 mm glass surfaces with microcontact printed stripes. Using an Olympus IX inverted microscope (Olympus, Hamburg, Germany) with a Delta Vision RT system (Applied Precision Inc., Issaquah, WA, USA), 25 fields of view (603.03 μm × 603.06 μm) were recorded every 10 min for at least 10 h using a 10×air objective lens (EC-PlanNeoFluor 10×/0.3 Ph1, Zeiss, Oberkochen, Germany) and detected with a cooled CCD camera (Photometrics, Kew, Australia). Image acquisition was performed with Resolve 3D (AppliedPrecision Inc.). Cell nuclei were tracked by the ImageJ plugin manual tracking. Thus, the velocity of the cells was analyzed and the net distances per hour were calculated and also summed up to determine the total path lengths of the cells.

### Statistic analysis

All experiments were performed in at least two technical repeats. Plots and histograms were generated with Microsoft Office Excel 2007, while box-plots were created with Prism 6 [version 6.04 (Trial), GraphPad]^2^. Values of the standard error of the mean (SEM) were calculated from the raw data and were used for error bars on graphs. Comparison between two sets of measures was performed using Mann–Whitney test (two-tailed, 95% confidence level: ***P < 0.001, **P < 0.01, *P < 0.1).

## Results

### BMP-2 is successfully immobilized on homogenous cFN-coated surfaces and is not released after immobilization

**Figure 2:**
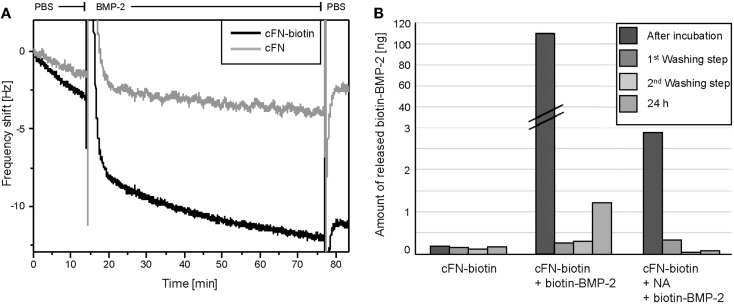
**(A)** Frequency shifts of F7 measured by QCM-D. Measurements of the adsorption of BMP-2 on QCM-D sensors coated with cFN-biotin (black) and cFN without biotinylation (gray) followed by the binding of NA and BMP-2-biotin. Here, the detail of the exchange from buffer to BMP-2-biotin and the following washing step with PBS is shown in the graph. Note the peaks represent the addition of the appropriate agent and the difference in frequency shift corresponding to a higher mass adsorption on the sensor with biotinylated cFN (black). **(B)** ELISA quantification of the release of BMP-2 from the incubated surfaces. cFN-biotin surfaces in absence of BMP-2 served as negative control. For the other two conditions, surfaces exposed to BMP-2-biotin with and without NA as cross-linker were used. The release was determined in the removed incubation solution as well as in the three washing solutions [first washing step, second washing step, third washing step (24 h)].

Prior to cell experiments, we characterized the surfaces by two different methods, namely QCM-D and ELISA. To directly monitor the binding of BMP-2 to cFN, we used QCM-D, which detects the subsequent adsorption of mass to a quartz crystal, resulting in a shift in the crystal’s resonance frequency. As already described above “BMP-2 is Successfully Immobilized on Homogenous cFN-Coated Surfaces and is Not Released After Immobilization,” cFN/cFN-biotin was stamped on the QCM-D crystal. After a washing step with PBS, NA was incubated on the crystal followed by another washing step. Finally, BMP-2-biotin was added to the system. Note the difference in adsorption of BMP-2 in absence and presence of biotinylated cFN (Figure 2A). This difference was especially visible during the washing step following the initial adsorption of the protein. Due to its unspecific interactions with materials, BMP-2 easily adsorbs to both surfaces but it is released after washing with PBS if NA is not bound to the biotinylated cFN. However, not all BMP-2 was removed by the washing step, suggesting a persisting interaction of BMP-2 with cFN at −2 Hz. In the presence of biotinylated cFN, a plateau was reached at −11 Hz after washing for several minutes, indicating the presence of a fraction of BMP-2 stably bound to the surface.

With ELISA, we assessed the amount of iBMP-2 by measuring the amount of the protein released in solution after 60 min of incubation and for each of the three washing steps (Figure 2B). Here, cFN/cFN-biotin was homogenously stamped on glass substrates and then incubated with NA and BMP-2-biotin. We derived the amount of surface iBMP-2 by subtracting the amount of protein detected in incubation and washing solutions from the initial amount of BMP-2 in solution. Figure 2B depicts the three conditions, namely cFN-biotin without NA and BMP-2, cFN-biotin with BMP-2 but without NA, and cFN-biotin with BMP-2 and NA. This corresponds to a surface concentration of iBMP-2, which was approximately 520 ng/cm^2^.

### Matrix-immobilized BMP-2 induces Smad 1/5 phosphorylation and prevents myotube formation in C2C12

**Figure 3:**
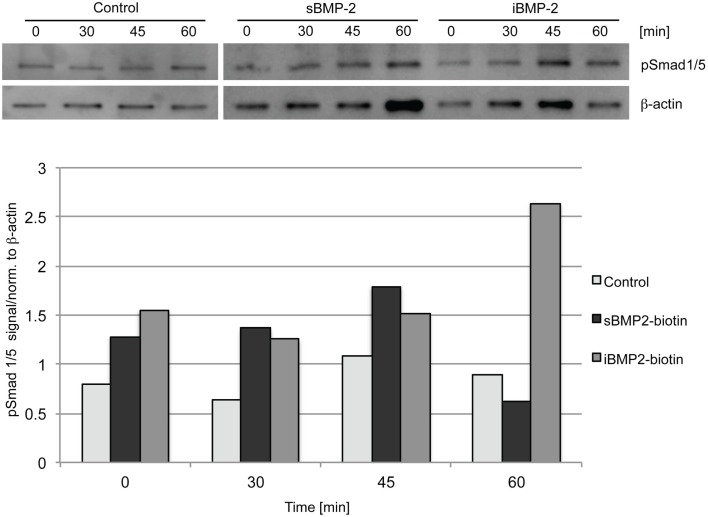
**Western blot of Smad 1/5 phosphorylation in C1C12 cells**. Serum-starved cells were seeded on the surfaces for 30 min (indicated as time point 0) and lysed after 30, 45, and 60 min. cFN surfaces serve as control, sBMP-2 indicates cFN surfaces where BMP-2 has been added to the media at the indicated durations. iBMP-2 indicates cFN surfaces with crosslinked biotinylated-BMP-2. Non-stimulated cells adhering to the control surfaces show basal Smad 1/5 phosphorylation signals, which are time-independent, whereas cells stimulated with sBMP-2 and iBMP-2 present pronounced Smad phosphorylation. β-actin served as loading control.

To investigate the bioactivity of iBMP-2, Smad 1/5 activation was evaluated by western blotting and indirect immunofluorescent staining. Cells were left to adhere on the surfaces for 30 min (indicated as time point 0) and then Smad 1/5 (pSmad 1/5) phosphorylation and nuclear translocation were monitored over time. The initial 30-min incubation time is necessary since addition of sBMP-2 is not sufficient to activate the Smad pathway in spreading cells, because of rapid depletion of the GF from the medium (data not shown). For the assessment of Smad phosphorylation, C2C12 myoblasts were seeded on surfaces coated with cFN in absence of BMP-2 (negative control), whereas cells incubated in presence of sBMP-2 in the media served as positive control. Cells were lysed after 0, 30, 45, and 60 min and the phosphorylation levels and kinetics of Smad 1/5 were determined by western blotting. Smad phosphorylation is detected only in cells exposed to sBMP-2 and iBMP-2 after 30 min, whereas only a faint band is present in the samples without BMP-2 (Figure 3). iBMP-2 is able to trigger Smad-dependent signaling and the levels of Smad phosphorylation are comparable to those observed when BMP-2 is added to the media (sBMP-2).

**Figure 4:**
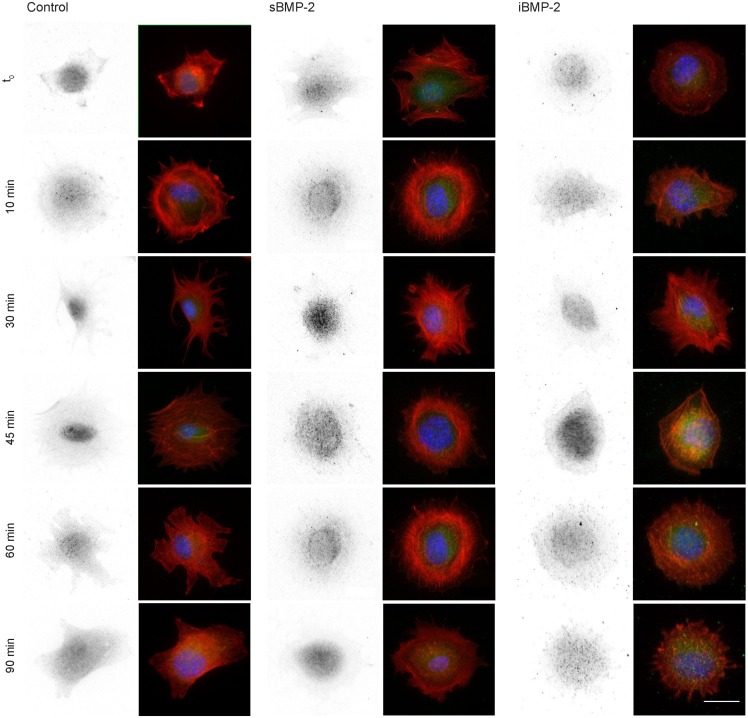
**Indirect immunofluorescence of pSmad 1/5 at different time points, from *t*_0_ to 90 min**. Cells were allowed to settle on the surface for 30 min (*t*_0_) to ensure proper adhesion. Note sBMP-2 was added to the medium at *t*_0_ whereas cells seeded on iBMP-2-surfaces were exposed upon surface contact. pSmad is depicted in the first column and appears green in the merged images, nuclei are stained with DAPI (blue), pSmad (green), and actin staining (red) were shown in a merged fluorescent channel (second column). Scale bar 20 μm.

**Figure 5:**
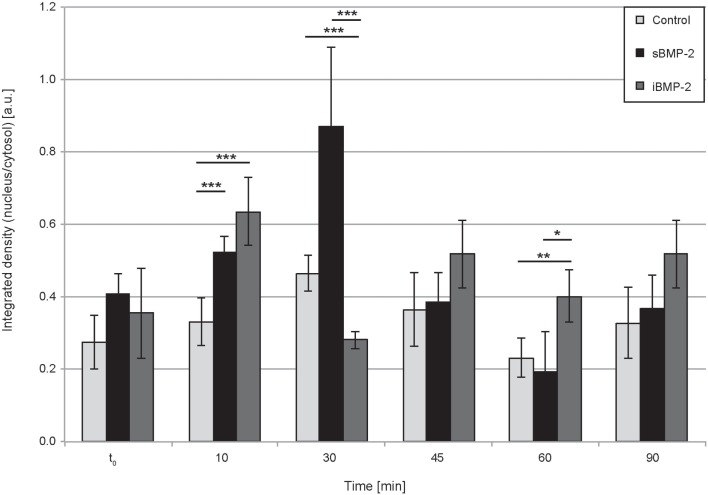
**Quantification of pSmad translocation**. Ratio of integrated densities for the pSmad signal in the cytosol and nucleus at the indicated time points. ***P < 0.001, **P < 0.01, *P < 0.1.

Next, we monitored the shuttling of pSmad 1/5 from the cytoplasm to the nucleus. Upon phosphorylation, Smad 1/5/8 forms the Smad complex, which acts as transcription factor in the nucleus. C2C12 cells were first allowed to adhere to the surface for 30 min and then fixed after additional 10, 30, 45, and 60 min time (Figure 4). The quantification of the shuttling of the active Smad complex from the cytoplasm to the nucleus shows a peak at 30 min in cells exposed to sBMP-2 that drops hereafter to control levels (Figure 5). The nuclear translocation of pSmad 1/5 in cells on iBMP-2 instead presents a different pattern, with an initial increase above the levels observed for the sBMP-2 at 10 min, followed by a drop at 30 min. It should be noted that cells seeded on iBMP-2 surfaces were exposed to BMP-2 upon surface contact. After 45 min, iBMP-2 leads again to an increased and sustained accumulation of nuclear pSmad in comparison to the control and the sBMP-2 groups. This increase could be explained by the progression of cell-surface interactions, i.e., cells are exposed to additional matrix-bound GFs.

**Figure 6:**
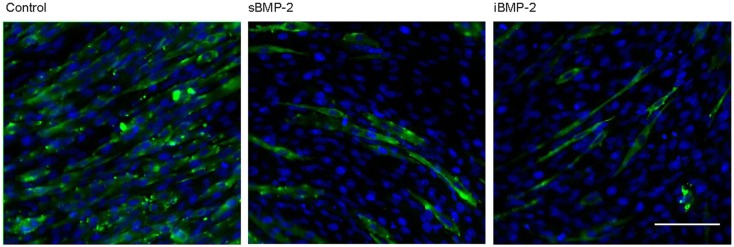
**Long-term response of C2C12 cells after 6 days in the presence of BMP-2**. Indirect immunofluorescence was employed to stain the myosin heavy subunit expressed in myotubes (green). The cell nuclei were stained with DAPI (blue). Myotube formation was suppressed by sBMP-2 and iBMP-2 but not for cultures of cells adhering to the control samples. Scale bar 100 μm.

C2C12 cells spontaneously fuse and form contractile multinucleated myotubes after few days in culture. In presence of osteoinductive GFs, such as BMP-2, this process is suppressed. Here, we investigated the influence of iBMP-2 on myotube formation in C2C12 cell cultured for 6 days on control surfaces (cFN only, without BMP-2), in presence of BMP-2 added to the culture media (sBMP-2) and on iBMP-2. MHC was stained by indirect immunofluorescence as myotube-specific marker. Figure 6 clearly depicts myotube formation in cells not exposed to BMP-2 as indicated by the green staining. Both sBMP-2 and iBMP-2 successfully suppress myotube formation, as can be seen in the reduced staining of MHC. Upon conjugation with biotin, we observed a slight decrease in the bioactivity of BMP-2 due to the biotinylation (see Figure S2 in Supplementary Material).

### Matrix-immobilized BMP-2 affects cell migration on microcontact printed structures

**Figure 7:**
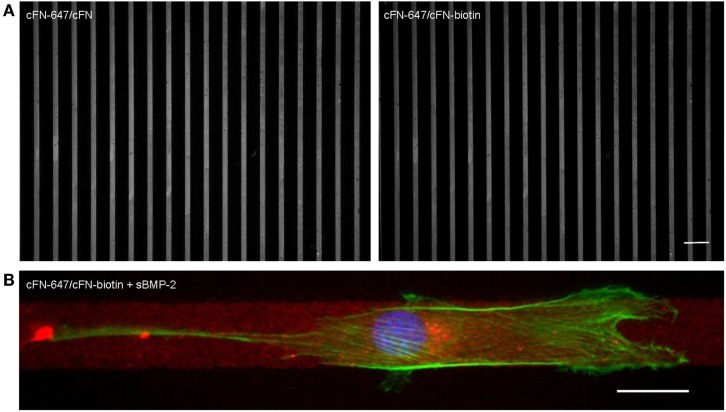
**(A) Cell adhesion on microcontact printed stripes of 20 μm width**. A mixture of fluorescently labeled cFN-647 and cFN (left), and cFN-647/cFN-biotin (right) was transferred to a glass slide previously activated by oxygen plasma treatment. The spacing between the stripes is 50 μm. Scale bar 100 μm. **(B) Single C2C12 cell migrating on 20 μm cFN-biotin stripes in the presence of sBMP-2**. The actin cytoskeleton was stained with phalloidin-TRITC (green) and the nucleus with DAPI (blue), whereas the cFN-647 stripe is labeled in red. Scale bar 20 μm.

For the investigation of cell migration in presence of iBMP-2, cFN was micropatterned in stripes, which served as adhesive and directional guidance. The dimensions of the stripes were chosen in accordance to the cell size and morphology; thus to ensure that only single cells would migrate in a directed fashion and would not bridge over several stripes, the stripes had a width of 20 μm and spacing between the stripes 50 μm. The surface between the cFN stripes was passivated with PLL–PEG to prevent unspecific cell adhesion to the glass. Figure 7A shows an example of a micropatterned glass surface. Fluorescently labeled cFN mixed with unconjugated cFN at a ratio of 1:1 is microcontact printed to visualize the integrity of the patterned stripes. The stripes were inspected after each preparation step and remained intact throughout the entire process (Figure S1 in Supplementary Material). C2C12 myoblasts are able to adhere to the cFN pattern and restricted to migrate on the stripes only, as can be seen in Figure 7B.

**Figure 8:**
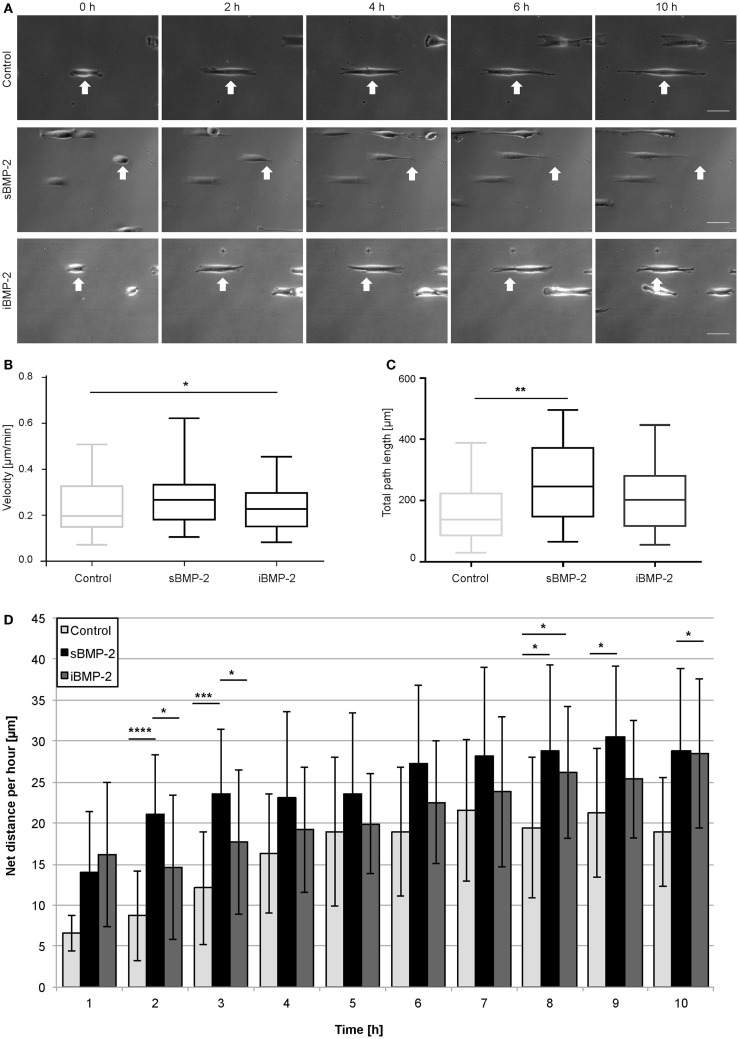
**C2C12 migration on microcontact printed stripes**. **(A)** Cells were cultured in serum conditions on 20 μm cFN-biotin stripes. Within the first 10 h, the migration behavior upon sBMP-2, iBMP-2, and no BMP-2 exposure is shown. All experiments were carried out in two technical repeats, with at least 25 cells per condition. Scale bar 50 μm. **(B)** Cell velocities upon different BMP-2 exposures on cFN-biotin stripes over a period of 10 h. *P < 0.1. Horizontal bars represent the median. **(C)** Averaged total path lengths of cells upon different BMP-2 exposures. **P < 0.01. Horizontal bars represent the median. **(D)** Averaged net distances of cells in 1 h intervals. ****P < 0.0001, ***P < 0.001, *P < 0.1.

As described in previous sections for the homogenously stamped surfaces, BMP-2-biotin was immobilized on cFN/cFN-biotin micropatterned stripes using NA as cross-linker. The surface in between the cFN stripes was passivated to prevent adhesion. As positive control, sBMP-2 was added to the cell media while as negative control only cFN stripes without addition sBMP-2 were used. Figure 8A shows a time course of cell migration along micropatterned stripes in the three different conditions, namely control, sBMP-2, and iBMP-2 (see also Video S1–S3 in Supplementary Material). The arrows indicate the starting point of one cell per condition over time. The velocities of the three conditions are depicted in Figure 8B as box-plots, where the median is displayed. Cells on all three conditions feature a similar velocity pattern with only slightly differing median values (control: 0.196 μm/min; sBMP-2: 0.265 μm/min; iBMP-2: 0.227 μm/min). When analyzing the steadiness of cell migration by calculating the net distances per hour, we found differences in the distance pattern covered by the cells on the three conditions. Figure 8D shows the migration profile divided into 1 h intervals. Cells on sBMP-2 and iBMP-2 display a similar preference to higher distances, especially at later time points. In contrast, in absence of BMP-2, cells show a rather constant behavior. Figure 8C summarizes the total path length of the cells as box-plot taking all net distances into account. Accordingly, cells upon BMP-2 exposure tend to cover larger distances, as can be seen at the varying median values (control: 137 μm; sBMP-2: 245 μm; iBMP-2: 202 μm).

## Discussion

The study presented here illustrates the immobilization of BMP-2, on cFN by employing the specific interaction between two biotinylated proteins and NA as cross-linker. The immobilized molecule retains its bioactivity since it activates short-term signaling pathways, such as the Smad-dependent pathway as well as long-term signaling pathways, such as suppression of myotube formation. The amount of conjugated linkers per BMP-2 molecule was assumed using structural information of BMP-2 by considering only accessible lysine residues (11 and 15), which do not interfere with receptor binding (Pohl et al., 2012). The less accessible and less favorable lysine residues (73, 76, 97, and 101) might be as well targeted during conjugation, although the orientation of BMP-2 onto cFN might be not essential for maintaining its activity.

After immobilization of the biotinylated BMP-2 and removal of protein excess, the release from the surface was determined by ELISA during two successive washing steps and after 24 h. The amount of BMP-2 released during the washing steps were below the detection limit. The methods currently used for quantification of GFs in solution allow detection as low as 0.25 ng. Thus, it cannot be excluded that small amounts of BMP-2 might be released but these are below the detection limit of the here employed ELISA.

Surface immobilization of BMP-2 via chemical linkers offers the advantage of having a sustained presentation while reducing the amount of the GF needed to trigger cell responses. Lagunas et al. introduced a system to generate BMP-2 gradients, with a ligand density ranging from 1.4 to 2.3 pmol/cm^2^ (Lagunas et al., 2010, 2013). Chemical surface gradients were established by hydrolysis of PMMA. Due to the strong electrostatic interactions of the BMP-2 molecule with biocompatible materials, other approaches rely on mere physisorption or embedding in films or gels such as PLL–HA as was shown by Crouzier et al. (2011) and Almodóvar et al. (2014). They attached polyelectrolyte multilayer films to substrates by alternating the deposition of PLL and hyaluronan. BMP-2 was loaded and entrapped within these films. The concentration of the entrapped BMP-2 was approximately 700 ng/cm^2^, while only superficial molecules of the multilayer are accessible for the cells.

Triggering Smad and non-Smad signaling pathways by iBMP-2 pinpoint that ligand–receptor interaction rather than cellular uptake of the GF is of particular importance. In fact, it has been shown that the initial interactions at the plasma membrane are sufficient for triggering Smad-dependent signaling (Bonor et al., 2012) and that ligand internalization is not necessary for such responses (Pohl et al., 2012). Smad signaling might occur independently of endocytosis involving clathrin-coated pit but rather require caveolin-mediated receptor internalization (Bonor et al., 2012). In the current study, we cannot exclude that the GF alone or the complex of GF–FN molecules are internalized over time. At the same time, it could be possible that activated receptors might traffic upon ligand recognition and binding, even without internalization of the ligand. However, the mode of presentation and delivery *in vivo* is still a matter of debate. Recently, binding sites for GFs belonging to the TGF-β superfamily were verified in several ECM glycoproteins. Also, other GF binding sites have been found in ECM proteins, such as collagen and FN (Wijelath et al., 2006; Hynes, 2009; Martino and Hubbell, 2010). *In vivo*, the ECM increases the GFs’ bioavailability by serving as a reservoir. GFs can be constitutively available or presented upon exogenous or cellular action. Cryptic binding sites are exposed upon injury or proteolytic processing leading to a rapid localized availability of GFs. Also, tension forces (Zhu and Clark, 2014) affect GF presentation. The juxtaposition with RGD-motives in the ECM facilitates integrin-mediated cell adhesion and directly links GF signaling to integrin signaling (Lin et al., 2011; Motegi et al., 2011; Fujita et al., 2012).

The mode of GF presentation is essential for medical applications. Amongst other issues, the amount of bioactive factors is a critical subject, since too high dosages bear the risks of side effects and are cost ineffective. To evaluate the minimal amount of BMP-2 necessary to trigger the desired cellular responses, BMP-2 has been immobilized on hexagonally ordered gold nanoparticle arrays with varying interparticle distances (Schwab et al., 2015). In this study, cells were exposed to the GF by sandwiching the BMP-2 bearing surfaces head first on the adherent cells. The cell interacts with the GF at their apical side. In contrast to this approach, we present simultaneously BMP-2 and FN, being both cues at the basal side of the cell. Such mode of presentation effectively triggered the Smad-dependent signaling as shown by Smad phosphorylation and nuclear shuttling of the Smad signaling complex as well as by suppression of myotube formation. The activation of the Smad-dependent signaling pathway as assessed here by Smad translocation into the nucleus depicted a strong peak for sBMP-2 at around 30 min followed by a decrease to control levels. iBMP-2 on the other hand exhibited overall lower values but sustained a higher activity level during the time of observation. This behavior might be explained by considering that sBMP-2 is only available for a short period of time whereas in the case of iBMP-2, the cells are constantly exposed to the GF while interacting with the surface.

Here, we also investigated the non-transcriptional effect of BMP-2 by assessing the influence of matrix-iBMP-2 on cell migration mediated by cytoskeletal rearrangements. We chose micropatterned stripes to restrict cell migration to only two directions. Previous studies have indicated that 1D topography can mimic many aspects of fibrillar-oriented 3D cell-derived ECMs. In both 1D and 3D, cell migration is a rapid and uniaxial process, whereby external physical stimuli in the form of linear topographical cues can regulate cell behavior (Doyle et al., 2009). On single 1D micropatterned lines, myoblasts can align, polarize, and migrate directionally (Ghibaudo et al., 2009). Thus, these type of patterned structures provide at the same time a simpler and more representative system than 2D systems for understanding 3D fibrillar matrix regulation of the dynamics of cell adhesion and signaling during cell migration (Cukierman et al., 2002). However, in the system we presented here, the line width of 20 μm is too large to observe quasi-3D migration patterns.

We show that during the first 4 h of observation, BMP-2 affects cell migration regardless of the mode of BMP-2 presentation. In fact, C2C12 cells tend to enhance their migratory behavior as compared to the control. This observation is in agreement with Hiepen et al. (2014), although we did not apply chemotactic gradients.

While guidance cues provided by physical and structural properties of the ECM have emerged as key parameters for directing cell migration, additional signals provided by gradients of soluble chemoattractants are still considered as leading factors for orchestrating cell movements (Tzvetkova-Chevolleau et al., 2008). Hiepen et al. (2014) demonstrated the importance of BMP-2-induced PI3K signaling for chemotaxis. Thus, the application of a linear BMP-2 gradient resulted in an overall gain in directionality of cell migration toward the source of BMP-2 as well as in an increase of the covered distance. However, the nature of receptor interaction and the mechanism determining polarity at the plasma membrane level still needs to be elucidated. To this end, our approach presented here on the immobilization of GFs to ECM components can be further developed by establishing GF gradients by microfluidics (Almodóvar et al., 2014).

## Conflict of Interest Statement

The authors declare that the research was conducted in the absence of any commercial or financial relationships that could be construed as a potential conflict of interest.

## Supplementary Material

The Supplementary Material for this article can be found online at http://journal.frontiersin.org/article/10.3389/fbioe.2015.00062

## Footnotes

^1^http://imagej.nih.gov/ij/

^2^http://www.graphpad.com/demos/
