# Supplementary material for: Matrix-Immobilized BMP-2 on Microcontact Printed Fibronectin as an in vitro Tool to Study BMP-Mediated Signaling and Cell Migration
Source: Front Bioeng Biotechnol. 2015 May 11;3:62. doi: 10.3389/fbioe.2015.00062 (PMC4426815; doi:10.3389/fbioe.2015.00062)
Supplement: Supplementary file 4 [file image_1.pdf]

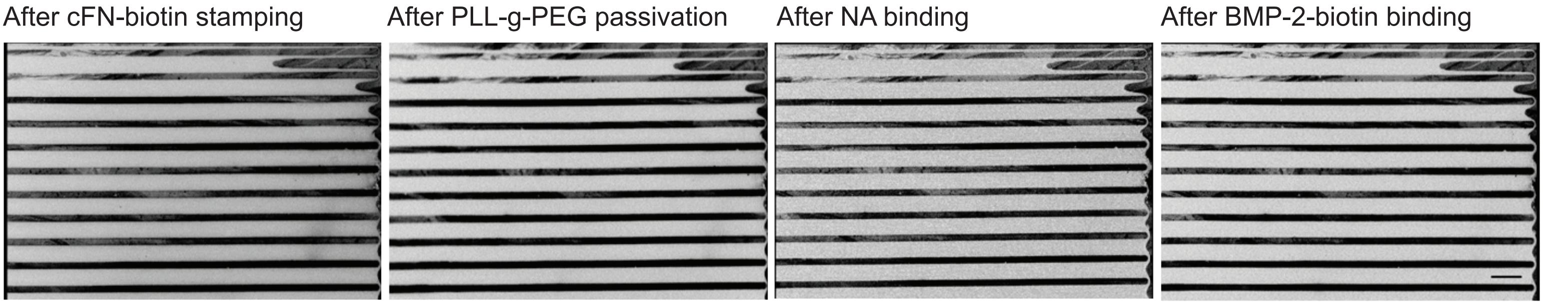

Control of the stripe quality of iBMP-2 surfaces after each step. The cFN-biotin/cFN-Atto647N stamping was followed by a passivation step with PLL-g-PEG and successive NA- and BMP-2-biotin incubation. The same position of the surface was monitored by fluorescence microscopy depicting no alteration of the patterned structure. Scale bar 100 µm.
