# Supplementary material for: Matrix-Immobilized BMP-2 on Microcontact Printed Fibronectin as an in vitro Tool to Study BMP-Mediated Signaling and Cell Migration
Source: Front Bioeng Biotechnol. 2015 May 11;3:62. doi: 10.3389/fbioe.2015.00062 (PMC4426815; doi:10.3389/fbioe.2015.00062)
Supplement: Supplementary file 5 [file image_2.pdf]

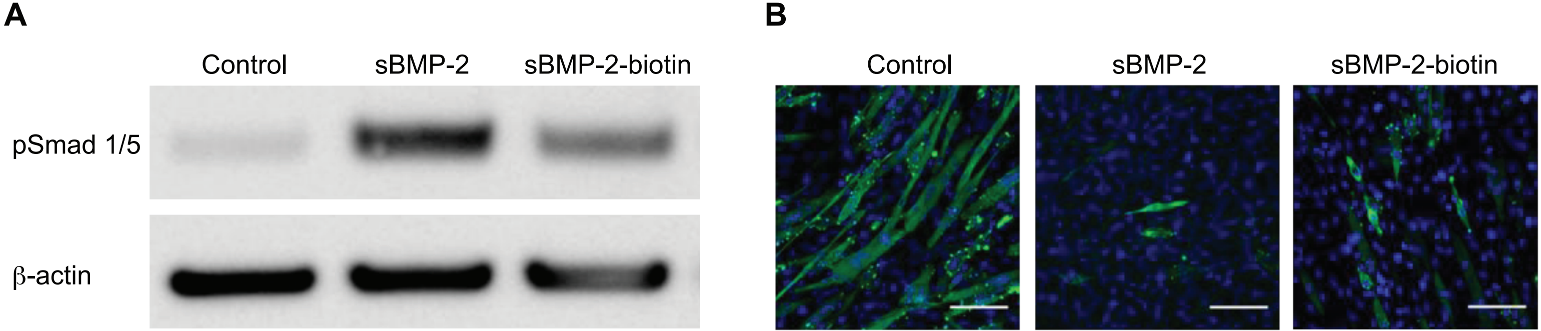

Short-and long-term responses of C2C12 to different soluble BMP-2 variants. (A) Short-term response: Smad phosphorylation of C2C12 cells exposed to soluble native and biotinylated BMP-2 at a concentration of 20 nM in comparison to control (no BMP-2). (B) Long-term response: Suppression of myotube formation. C2C12 cells were cultured for 6 days in the presence of either native or biotinylated BMP-2. No BMP-2 in the system was used as control. MHC staining is shown in green and DAPI staining in blue. Scale bar 100 μm.
